# Supplementary material for: Analytical Validation of the Cxbladder® Triage Plus Assay for Risk Stratification of Hematuria Patients for Urothelial Carcinoma
Source: Diagnostics (Basel). 2025 Jul 8;15(14):1739. doi: 10.3390/diagnostics15141739 (PMC12293762; doi:10.3390/diagnostics15141739)
Supplement: Supplementary file 1 [file diagnostics-15-01739-s001.zip › diagnostics-3648837-supplementary.pdf]

Supplementary Materials

Analytical Validation of the Cxbladder® Triage Plus Assay for Risk Stratification of Hematuria Patients for Urothelial Carcinoma

Justin C. Harvey [1,*], David Fletcher [2], Charles W. Ellen [1], Megan Colonval [1], Jody A. Hazlett [1], Xin Zhou [1] and Jordan M. Newell [3]

Table S1. Demographics of patients with hematuria who were included in the algorithm development dataset (N=987).

Characteristic	Overall (N=987)
Sex, n (%)	
Female	444 (45.0)
Male	543 (55.0)
Age group, n (%)	
18–30 years	15 (1.5)
30–40 years	39 (4.0)
40–50 years	109 (11.0)
50–60 years	226 (22.9)
60–70 years	336 (34.0)
70–80 years	201 (20.4)
80–90 years	58 (5.9)
≥90 years	3 (0.3)
Hematuria status, n (%)	
Gross hematuria	605 (61.3)
Microhematuria	382 (38.7)
Pathology confirmed UC diagnosis, n (%)	
Negative	909 (92.1)
Positive	78 (7.9)
HG [a] or Cis	52 (5.3)
LG [b]	26 (2.6)

[a] Defined as high grade or stage >Ta.

[b] Defined as low grade and stage Ta.

Cis, carcinoma in situ; HG, high grade; LG, low grade; UC, urothelial carcinoma.

Table S2. Confusion matrix of Cxbladder Triage Plus algorithm at the upper and lower threshold compared with tumor status confirmed by pathology in the algorithm development dataset (N=987).

**Tumor status**	**Score threshold 0.15**		**Score threshold 0.54**		**Total**
	**Negative**	**Positive**	**Negative**	**Positive**	
Negative	825	84	893	16	909
Positive	5	73	31	47	78
HG [a] or Cis	2	50	17	35	52
LG [b]	3	23	14	12	26
**Total**	**830**	**157**	**924**	**63**	**987**

[a] Defined as high grade or stage >Ta.

[b] Defined as low grade and stage Ta.

Cis, carcinoma in situ; HG, high grade; LG, low grade.

Table S3. Analytical specificity of Cxbladder Triage Plus with control samples when mixed with process-derived interfering substances.

Substance	Tolerable percentage of inhibitor (%) [a]	Tolerable volume per 64 μL elution (μL)	Subjective risk appraisal
Absolute ethanol	4	2.56	Very low
Acetone	10	6.4	Very low
Cxbladder stabilizing reagent	1	0.64	Very low
MagMAX wash buffer	2	1.28	Very low
MagMAX magnetic beads	5	3.2	Very low

[a] Percentage of total ddPCR volume.

ddPCR, droplet-digital polymerase chain reaction.

Table S4. Lot-to-lot Cxbladder reagent variance for the six DNA single-nucleotide variants of *FGFR3* and *TERT* in Cxbladder Triage Plus.

**SNV**	**Mutant fraction**							
	**Magnetic beads lot A [a] + wash buffer lot A [b]**		**Magnetic beads lot A [a] + wash buffer lot B [c]**		**Magnetic beads lot B [d] + wash buffer lot B [c]**		**Magnetic beads lot C [e] + wash buffer lot C [f]**	
	**Mean (SD)**	**CV%**	**Mean (SD)**	**CV%**	**Mean (SD)**	**CV%**	**Mean (SD)**	**CV%**
FGFR3								
HEC [g]	0.0872 (0.0022)	2.53	0.0868 (0.0012)	1.39	0.0863 (0.0021)	2.48	0.0856 (0.0010)	1.14
LEC [h]	0.0072 (0.0009)	13.10	0.0072 (0.0011)	15.44	0.0075 (0.0012)	16.49	0.0085 (0.0015)	17.64
TERT								
HEC [g]	0.1024 (0.0018)	1.74	0.1026 (0.0013)	1.24	0.1020 (0.0009)	0.84	0.1018 (0.0014)	1.33
LEC [h]	0.0105 (0.0016)	15.10	0.0099 (0.0032)	32.15	0.0126 (0.0014)	11.46	0.0128 (0.0022)	17.25

[a] MagMAX magnetic beads lot BD2206339.

[b] MagMAX wash buffer lot WB2405057.

[c] MagMAX wash buffer lot WB2405056.

[d] MagMAX magnetic beads lot BD2206338.

[e] MagMAX magnetic beads lot BD2302342.

[f] MagMAX wash buffer lot WB2312054.

[g] DNA concentration of ~1×10^6 copies/μL and mutant:WT ratio of 1:10.

[h] DNA concentration of ~1×10^4 copies/μL and mutant:WT ratio of 1:200.

CV%, coefficient of variation; *FGFR3*, fibroblast growth factor receptor 3; HEC, high-extraction control; LEC, low-extraction control; SD, standard deviation; SNV, single-nucleotide variant; *TERT*, telomerase reverse transcriptase.
